# Supplementary material for: Epidemiological research on rare diseases using large-scale online search queries and reported case data
Source: Orphanet J Rare Dis. 2023 Aug 9;18:236. doi: 10.1186/s13023-023-02839-7 (PMC10411025; doi:10.1186/s13023-023-02839-7)
Supplement: Supplementary file 1 — Supplementary Material 1 [file 13023_2023_2839_MOESM1_ESM.docx]

**Supplementary table 1. Keywords for 120 rare diseases**

| **Disease name** | **Keywords** |
| --- | --- |
| 21-Hydroxylase Deficiency | 21-羟化酶缺乏症，21羟化酶缺乏症，二十一羟化酶缺乏症，21-hydroxylase deficiency，21 hydroxyulase deficiency，21-ohd |
| Albinism | 白化病，albinism |
| Alport Syndrome | alport综合征，奥尔伯特综合征，奥尔伯特氏综合征，奥尔波特综合征，奥尔波特氏综合征，阿尔伯特综合征，阿尔伯特氏综合征，阿尔波特综合征，阿尔波特氏综合征，眼-耳-肾综合征，眼耳肾综合征，遗传性肾炎，alport syndrome |
| Amyotrophic Lateral Sclerosis | 肌萎缩侧索硬化，肌萎缩性侧索硬化，肌肉萎缩侧索硬化，肌肉萎缩性侧索硬化，肌肉萎缩脊髓侧索硬化，肌肉萎缩性脊髓侧索硬化，运动神经元病，卢伽雷病，夏科病，渐冻人症，渐冻人，lou gehrig病，charcot病，amyotrophic lateral sclerosis |
| Angelman Syndrome | angelman氏症候群，天使综合征，天使人综合征，快乐木偶综合征，安格曼综合征，angelman syndrome |
| Arginase Deficiency | 精氨酸酶缺乏症，精氨酸血症，高精氨酸血症，arginase deficiency，argininemia |
| Asphyxiating Thoracic Dystrophy | 热纳综合征，窒息性胸腔失养症，jeune综合征，asphyxiating thoracic dystrophy，jeune syndrome |
| Atypical Hemolytic Uremic Syndrome | 非典型溶血性尿毒症，非典型溶血性尿毒症综合征，atypical hemolytic uremic syndrome，ahus |
| Autoimmune Encephalitis | 自身免疫性脑炎，autoimmune encephalitis |
| Autoimmune Hypophysitis | 自身免疫性垂体炎，autoimmune hypophysitis |
| Autoimmune Insulin Receptopathy | 自身免疫性胰岛素受体病，b型胰岛素抵抗，autoimmune insulin receptopathy， type b insulin resistance |
| β-Ketothiolase Deficiency | β-酮硫解酶缺乏症，β酮硫解酶缺乏症，线粒体乙酰乙酰基辅酶a硫解酶缺乏症，β-ketothiolase deficiency，β ketothiolase deficiency，beta-ketothiolase deficiency，beta ketothiolase deficiency，mitochondrial acetoacetyl-coa thiolase (3- oxothiolase) deficiency，mitochondrial acetoacetyl coa thiolase (3 oxothiolase) deficiency |
| Biotinidase Deficiency | 生物素酶缺乏症，biotinidase deficiency |
| Cardiac Ion Channelopathies | 心脏离子通道病，遗传性原发性心律失常综合征，cardiac ion channelopathies |
| Castleman Disease | castleman病，血管滤泡性淋巴结增生症，巨大淋巴结增生症，castleman disease |
| Charcot-Marie-Tooth Disease | 腓骨肌萎缩症，遗传性运动感觉神经病，charcot-marie-tooth disease，charcot marie tooth disease，peronial myoatrophy，hereditary motor and sensory neuropathy |
| Citrullinemia | 瓜氨酸血症，citrullinemia |
| Congenital Adrenal Hypoplasia | 先天性肾上腺发育不良，先天性x-连锁肾上腺发育不良，先天性x连锁肾上腺发育不良，先天性肾上腺皮质功能减退，先天性肾上腺皮质功能减退症，congenital adrenal hypoplasia，adrenal hypoplasia congenita |
| Congenital Hyperinsulinemic Hypoglycemia | 先天性高胰岛素性低血糖血症，婴儿持续性高胰岛素血症性低血糖症，congenital hyperinsulinemic hypoglycemia |
| Congenital Myasthenic Syndrome | 先天性肌无力综合征，congenital myasthenic syndrome |
| Congenital Myotonia | 先天性肌强直，非营养不良性肌强直综合征，congenital myotonia |
| Congenital Scoliosis | 先天性脊柱侧弯，先天性脊柱侧凸，congenital scoliosis |
| Coronary Artery Ectasia | 冠状动脉扩张病，coronary artery ectasia |
| Diamond-Blackfan Anemia | 先天性纯红细胞再生障碍性贫血，diamond-blackfan贫血，diamond blackfan贫血，diamond-blackfan综合征，diamond blackfan综合征，diamond-blackfan anemia，diamond blackfan anemia |
| Erdheim-Chester Disease | erdheim-chester病，erdheim chester病，脂质肉芽肿病，脂质肉芽肿瘤样增生病，erdheim-chester disease，erdheim chester disease，lipogranulomatosis |
| Fabry Disease | 法布雷病，法布里病， fabry病，安德森-法布里病，安德森法布里病，anderson-fabry病，anderson fabry病，α-半乳糖苷酶a缺乏病，α半乳糖苷酶a缺乏病，血管角质瘤综合征，fabry disease，anderson-fabry disease，anderson fabry disease，α-galactosidase a deficiency，α galactosidase a deficiency，alpha-galactosidase a deficiency，alpha galactosidase a deficiency |
| Familial Mediterranean Fever | 家族性地中海热，familial mediterranean fever |
| Fanconi Anemia | 范可尼贫血，fanconi贫血，fanconi anemia |
| Galactosemia | 半乳糖血症，galactosemia |
| Gaucher Disease | 戈谢病，gaucher病，葡糖脑苷脂病，gaucher disease |
| Generalized Myasthenia Gravis | 全身型重症肌无力，generalized myasthenia gravis |
| Gitelman Syndrome | gitelman综合征，吉特曼综合征，家族性低钾低镁血症，gitelman syndrome，familial hypokalemia-hypomagnesemia，familial hypokalemia hypomagnesemia |
| Glutaric Acidemia Type I | 戊二酸血症1型，戊二酸血症ⅰ型，戊二酸血症一型，glutaric acidemia typeⅰ |
| Glycogen Storage Disease（Type I，II） | 糖原累积病（i型、ⅱ型），糖原累积病（一型、二型），糖原累积病（1型、2型），糖原贮积症（i型、ⅱ型），糖原贮积症（一型、二型），糖原贮积症（1型、2型），i型糖原累积病，一型糖原累积病，1型糖原累积病，ⅰ型糖原贮积症，一型糖原贮积症，1型糖原贮积症，糖原累积病i型，糖原累积病一型，糖原累积病1型，ii型糖原累积病，二型糖原累积病，2型糖原累积病，ii型糖原贮积症，二型糖原贮积症，2型糖原贮积症，糖原累积病ii型，糖原累积病二型，糖原累积病2型，glycogen storage disease（type i, ii） |
| Hemophilia | 血友病，hemophilia |
| Hepatolenticular Degeneration | 肝豆状核变性，wilson病，威尔逊病，hepatolenticular degeneration，wilson disease |
| Hereditary Angioedema | 遗传性血管性水肿，遗传性血管神经性水肿，hereditary angioedema |
| Hereditary Epidermolysis Bullosa | 遗传性大疱性表皮松解症，hereditary epidermolysis bullosa |
| Hereditary Fructose Intolerance | 遗传性果糖不耐受症，hereditary fructose intolerance |
| Hereditary Hypomagnesemia | 遗传性低镁血症，家族性低镁血症，家族性肾性低镁，hereditary hypomagnesemia |
| Hereditary Multi-infarct Dementia | 遗传性多发脑梗死性痴呆，常染色体显性遗传性脑动脉病伴皮质下梗死和白质脑病，hereditary multi-infarct dementia，hereditary multi infarct dementia |
| Hereditary Spastic Paraplegia | 遗传性痉挛性截瘫，家族性痉挛性截瘫，strumpell-lorrain病，strumpell lorrain病，hereditary spastic paraplegia |
| Holocarboxylase Synthetase Deficiency | 全羧化酶合成酶缺乏症，holocarboxylase synthetase deficiency |
| Homozygous Familial Hypercholesterolemia | 纯合子家族性高胆固醇血症，homozygous familial hypercholesterolemia |
| Huntington’s Disease | 亨廷顿舞蹈病，亨廷顿病，慢性进行性舞蹈病，huntington’s disease |
| Hyperornithinaemia-Hyperammonaemia-Homocitrullinuria Syndrome | hhh综合征，高鸟氨酸血症-高氨血症-同型瓜氨酸尿症综合征，高鸟氨酸血症高氨血症同型瓜氨酸尿症综合征，鸟氨酸转移酶缺乏症，hyperornithinaemia-hyperammonaemia-homocitrullinuria syndrome，hyperornithinaemia hyperammonaemia homocitrullinuria syndrome |
| Hyperphenylalaninemia | 高苯丙氨酸血症，hyperphenylalaninemia |
| Hypophosphatasia | 低碱性磷酸酶血症，低磷酸酶血症，hypophosphatasia |
| Hypophosphatemic Rickets | 低磷性佝偻病，低磷血症性佝偻病，肾性低磷血症性佝偻病，家族性低磷血症，hypophosphatemic rickets |
| Idiopathic Cardiomyopathy | 特发性心肌病，idiopathic cardiomyopathy |
| Idiopathic Hypogonadotropic Hypogonadism | 特发性低促性腺激素性性腺功能减退症，idiopathic hypogonadotropic hypogonadism |
| Idiopathic Pulmonary Arterial Hypertension | 特发性肺动脉高压，原发性肺动脉高压，idiopathic pulmonary arterial hypertension，primary pulmonary arterial hypertension，ipah |
| Idiopathic Pulmonary Fibrosis | 特发性肺纤维化，idiopathic pulmonary fibrosis，idiopathic fibrosis of the lung |
| IgG4 Related Disease | igg4相关性疾病，igg4 related disease，igg4-related disease，igg4-rd |
| Inborn Errors of Bile Acid Synthesis | 先天性胆汁酸合成障碍，inborn errors of bile acid synthesis，iebas |
| Isovaleric Acidemia | 异戊酸血症，异戊酸尿症，异戊酰辅酶a脱氢酶缺乏症，isovaleric acidemia |
| Kallmann Syndrome | 卡尔曼综合征，卡尔曼氏综合征，kallmann syndrome |
| Langerhans Cell Histiocytosis | 朗格汉斯组织细胞增生症，langerhans细胞组织增生症，hand-schüller-christian病，hand-schuller-christian病，hand schüller christian病，hand schuller christian病，组织细胞增生症x，letterer-siwe病，letterer siwe病，嗜酸性肉芽肿，langerhans cell histiocytosis |
| Laron Syndrome | 莱伦氏综合征，原发性生长激素不敏感综合征，生长激素迟钝症候群，laron syndrome，primary growth hormone insensitivity syndrome |
| Leber Hereditary Optic Neuropathy | leber遗传性视神经病变，leber hereditary optic neuropathy |
| Long Chain 3-hydroxyacyl-CoA Dehydrogenase Deficiency | 长链3-羟酰基辅酶a脱氢酶缺乏症，长链3羟酰基辅酶a脱氢酶缺乏症，long chain 3 hydroxyacyl coa dehydrogenase deficiency，long chain 3-hydroxyacyl-coa dehydrogenase deficiency，lchadd |
| Lymphangioleiomyomatosis | 淋巴管肌瘤病，淋巴管平滑肌瘤病，lymphangioleiomyomatosis |
| Lysinuric Protein Intolerance | 赖氨酸尿蛋白不耐受症，阳离子氨基酸尿症，lysinuric protein intolerance |
| Lysosomal Acid Lipase Deficiency | 溶酶体酸性脂肪酶缺乏症，沃尔曼病，wolman病，lal缺乏症，lysosomal acid lipase deficiency |
| Maple Syrup Urine Disease | 枫糖尿症，槭糖尿病，支链ɑ-酮酸脱氢酶缺乏症，支链ɑ酮酸脱氢酶缺乏症，支链酮酸尿症，maple syrup urine disease |
| Marfan Syndrome | 马凡综合征，马凡氏综合征，马方综合征，马方氏综合征，蜘蛛指综合征，marfan syndrome |
| McCune-Albright Syndrome | mccune-albright综合征，mccune albright综合征，多发性骨纤维发育不良伴性早熟综合征，mccune-albright syndrome，mccune albright syndrome |
| Medium Chain Acyl-CoA Dehydrogenase Deficiency | 中链酰基辅酶a脱氢酶缺乏症，medium chain acyl-coa dehydrogenase deficiency，medium chain acyl coa dehydrogenase deficiency |
| Methylmalonic Acidemia | 甲基丙二酸血症，甲基丙2酸血症，甲基丙二酸尿症，甲基丙2酸尿症，methylmalonic acidemia，methylmalonic aciduria |
| Mitochondrial Encephalomyopathy | 线粒体脑肌病，mitochondrial encephalomyopathy |
| Mucopolysaccharidosis | 黏多糖贮积症，粘多糖症，粘多糖病，mucopolysaccharidosis |
| Multifocal Motor Neuropathy | 多灶性运动神经病，多灶性脱髓鞘性运动神经病，multifocal motor neuropathy |
| Multiple Acyl-CoA Dehydrogenase Deficiency | 多种酰基辅酶a脱氢酶缺乏症，戊二酸血症2型，戊二酸血症ⅱ型，戊二酸血症二型，multiple acyl-coa dehydrogenase deficiency，multiple acyl coa dehydrogenase deficiency，glutaric acidemia ii |
| Multiple Sclerosis | 多发性硬化，multiple sclerosis |
| Multiple System Atrophy | 多系统萎缩，散发性橄榄体桥脑小脑萎缩，橄榄脑桥小脑萎缩，纹状体黑质变性，shy-drager综合征，shy drager综合征，multiple system atrophy，olivoponto-cerebellar atrophy，olivoponto cerebellar atrophy，striatonigral degeneration |
| Myotonic Dystrophy | 肌强直性营养不良，强直性肌营养不良，萎缩性肌强直症，myotonic dystrophy |
| N-acetylglutamate Synthase Deficiency | n-乙酰谷氨酸合成酶缺乏症，n乙酰谷氨酸合成酶缺乏症，n-acetylglutamate synthase deficiency，n acetylglutamate synthase deficiency |
| Neonatal Diabetes Mellitus | 新生儿糖尿病，neonatal diabetes mellitus |
| Neuromyelitis Optica | 视神经脊髓炎，devic病，德维克病，neuromyelitis optica，optical neuromyelitis，devic's disease |
| Niemann-Pick Disease | 尼曼匹克病，尼曼匹克氏病，鞘磷脂胆固醇脂沉积症，鞘磷脂沉积病，niemann-pick disease，niemann pick disease |
| Nonsyndromic Deafness | 非综合征性耳聋，nonsyndromic deafness，nonsyndromic hearing loss |
| Noonan Syndrome | noonan综合征，努南综合征，努南氏症候群，noonan syndrome |
| Ornithine Transcarbamylase Deficiency | 鸟氨酸氨甲酰基转移酶缺乏症，高氨血症ⅱ型，高氨血症二型，高氨血症2型，ornithine transcarbamylase deficiency |
| Osteogenesis Imperfecta | 成骨不全症，脆骨病，原发性骨脆症，骨膜发育不良，玻璃骨，osteogenesis imperfecta，fragililisossium，fragililis ossium，periosteal dysplasia |
| Parkinson’s Disease (Young-onset, Early-onset) | 帕金森病（青年型、早发型），青年型帕金森病，帕金森病（青年型），早发型帕金森病，帕金森病（早发型），parkinson’s disease (young-onset, early-onset)，young-onset parkinson’s disease，young onset parkinson’s disease，early-onset parkinson’s disease，early onset parkinson’s disease |
| Paroxysmal Nocturnal Hemoglobinuria | 阵发性睡眠性血红蛋白尿，阵发性睡眠性血红蛋白尿症，夜间睡眠性血红蛋白尿，paroxysmal nocturnal hemoglobinuria |
| Peutz-Jeghers Syndrome | 黑斑息肉综合征，黑斑息肉病，家族性黏膜皮肤色素沉着胃肠道息肉病，波伊茨-耶格综合征，波伊茨耶格综合征，珀茨-杰格斯综合征，珀茨杰格斯综合征，pj综合征，peutz-jeghers syndrome，peutz jeghers syndrome |
| Phenylketonuria | 苯丙酮尿症，苯酮尿症，phenylketonuria |
| POEMS Syndrome | poems综合征，crow-fukase综合征，crow fukase综合征，takatsuki综合征，poems syndrome |
| Porphyria | 卟啉病，血卟啉病，血紫质病，紫质病，porphyria |
| Prader-Willi Syndrome | prader-willi综合征，prader willi综合征，小胖威利综合征，小胖威利症，普拉德-威利综合征，普拉德威利综合征，肌张力低下-智能障碍-性腺发育滞后-肥胖综合征，肌张力低下智能障碍性腺发育滞后肥胖综合征，低肌张力-低智力-性腺发育低下-肥胖综合征，低肌张力低智力性腺发育低下肥胖综合征，prader-willi syndrome，prader willi syndrome |
| Primary Carnitine Deficiency | 原发性肉碱缺乏症，肉碱摄取障碍，肉碱转运障碍，primary carnitine deficiency |
| Primary Combined Immunodeficiency | 原发性联合免疫缺陷，原发性联合免疫缺陷病，联合免疫缺陷，primary combined immunodeficiency |
| Primary Hereditary Dystonia | 原发性遗传性肌张力不全，原发性遗传性肌张力障碍，primary hereditary dystonia |
| Primary Light Chain Amyloidosis | 原发性轻链型淀粉样变，primary light chain amyloidosis |
| Progressive Familial Intrahepatic Cholestasis | 进行性家族性肝内胆汁淤积症，progressive familial intrahepatic cholestasis |
| Progressive Muscular Dystrophy | 进行性肌营养不良，progressive muscular dystrophy |
| Propionic Acidemia | 丙酸血症，丙酸尿症，propionic acidemia |
| Pulmonary Alveolar Proteinosis | 肺泡蛋白沉积症，肺泡磷脂沉着症，rosen-castleman-liebow综合征，rosen castleman liebow综合征，pulmonary alveolar proteinosis |
| Pulmonary Cystic Fibrosis | 肺囊性纤维化，pulmonary cystic fibrosis，cystic fibrosis of the lung |
| Retinitis Pigmentosa | 视网膜色素变性，毯层视网膜变性，retinitis pigmentosa |
| Retinoblastoma | 视网膜母细胞瘤，retinoblastoma |
| Severe Congenital Neutropenia | 重症先天性粒细胞缺乏症，severe congenital neutropenia |
| Severe Myoclonic Epilepsy in Infancy | 婴儿严重肌阵挛性癫痫，dravet综合征，severe myoclonic epilepsy in infancy |
| Sickle Cell Disease | 镰刀型细胞贫血病，镰刀型细胞贫血症，镰刀状细胞型贫血，镰状细胞贫血，sickle cell disease |
| Silver-Russell Syndrome | silver-russell综合征，silver russell综合征，silver-russell矮小症，silver russell矮小症，silver-russell syndrome，silver russell syndrome |
| Sitosterolemia | 谷固醇血症，植物固醇血症，sitosterolemia |
| Spinal Bulbar Muscular Atrophy | 脊髓延髓肌萎缩症，肯尼迪病，spinal bulbar muscular atrophy |
| Spinal Muscular Atrophy | 脊髓性肌萎缩症，进行性脊髓性肌萎缩症，脊髓性肌萎缩，脊肌萎缩症，spinal muscular atrophy |
| Spinocerebellar Ataxia | 脊髓小脑性共济失调，spinocerebellar ataxia |
| Systemic Sclerosis | 系统性硬化症，硬皮病，systemic sclerosis |
| Tetrahydrobiopterin Deficiency | 四氢生物蝶呤缺乏症，异型苯丙酮尿症，tetrahydrobiopterin deficiency |
| Tuberous Sclerosis Complex | 结节性硬化症，bourneville病，tuberous sclerosis complex |
| Tyrosinemia | 原发性酪氨酸血症，tyrosinemia |
| Very Long Chain Acyl-CoA Dehydrogenase Deficiency | 极长链酰基辅酶a脱氢酶缺乏症，very long chain acyl-coa dehydrogenase deficiency，very long chain acyl coa dehydrogenase deficiency，vlcadd |
| Williams Syndrome | 威廉姆斯综合征，williams综合征，williams-beuren综合征，williams beuren综合征，williams syndrome，williams-beuren syndrome，williams beuren syndrome |
| Wiskott-Aldrich Syndrome | 湿疹血小板减少伴免疫缺陷综合征，wiskott-aldrich综合征，wiskott aldrich综合征，wiskott-aldrich syndrome，wiskott aldrich syndrome |
| X-linked Adrenoleukodystrophy | x-连锁肾上腺脑白质营养不良，x连锁肾上腺脑白质营养不良，肾上腺脑白质营养不良，x-linked adrenoleukodystrophy，x linked adrenoleukodystrophy，x-ald |
| X-linked Agammaglobulinemia | x-连锁无丙种球蛋白血症，x连锁无丙种球蛋白血症，先天性无丙种球蛋白血症，bruton病，bruton综合征，x-linked agammaglobulinemia，x linked agammaglobulinemia |
| X-linked Lymphoproliferative Disease | x-连锁淋巴增生症，x连锁淋巴增生症，x-连锁淋巴组织增生综合征，x连锁淋巴组织增生综合征，x-连锁淋巴细胞异常增生症，x连锁淋巴细胞异常增生症，x-连锁淋巴组织增生性疾病，x连锁淋巴组织增生性疾病，x-连锁隐性进行性联合变异免疫缺陷病，x连锁隐性进行性联合变异免疫缺陷病，duncan病，x-linked lymphoproliferative disease，x linked lymphoproliferative disease，x-linked recessive progressive combined variable immune deficiency，x linked recessive progressive combined variable immune deficiency |
