# Supplementary material for: Epidemiological research on rare diseases using large-scale online search queries and reported case data
Source: Orphanet J Rare Dis. 2023 Aug 9;18:236. doi: 10.1186/s13023-023-02839-7 (PMC10411025; doi:10.1186/s13023-023-02839-7)
Supplement: Supplementary file 3 — Supplementary Material 3 [file 13023_2023_2839_MOESM3_ESM.docx]

**Epidemiological research on rare diseases using large-scale online search queries and reported case data**

**Authors full names:**

Lei Zhang^1^, Ye Jin^2^, Jiayu Li^3^, Zhiyu He^3^, Dingding Zhang^2^, Min Zhang^3^*, Shuyang Zhang^4^*

Lei Zhang, Ye Jin and Jiayu Li contribute equally to this manuscript.

**Affiliations:**

^1^ Department of Nephrology, State Key Laboratory of Complex Severe and Rare Diseases, Peking Union Medical College Hospital, Chinese Academy of Medical Science and Peking Union Medical College, Beijing, China.

^2^ Department of Medical Research Center, State Key Laboratory of Complex Severe and Rare Diseases, Peking Union Medical College Hospital, Chinese Academy of Medical Science and Peking Union Medical College, Beijing, China.

^3^ Department of Computer Science and Technology, Tsinghua University, Beijing, China.

^4^ Department of Cardiology, State Key Laboratory of Complex Severe and Rare Diseases, Peking Union Medical College Hospital, Chinese Academy of Medical Science and Peking Union Medical College, Beijing, China.

**Corresponding author:**

Min Zhang

Department of Computer Science and Technology, Tsinghua University, Beijing, 100084, China,

E-mail: z-m@tsinghua.edu.cn

Shuyang Zhang

Department of Cardiology, State Key Laboratory of Complex Severe and Rare Diseases, Peking Union Medical College Hospital, Beijing, 100730, China,

E-mail: zhangsy@nrdrs.org

**Abstract**

**Background:** Rare diseases have become a major public health concern worldwide. However, detailed epidemiological data are lacking. With the development of the Internet, search queries have played an important role in disease surveillance. In this study, we explored a new method for the epidemiological research on rare diseases, using large-scale online search queries and reported case data. We distilled search logs related to rare diseases nationwide from 2016 to 2019. The case data were obtained from China’s national database of rare diseases during the same period.

**Results:** A total of 120 rare diseases were included in this study. From 2016 to 2019, the number of patients with rare diseases estimated using search data and those obtained from the case database showed an increasing trend. Rare diseases can be ranked by the number of search estimated patients and reported patients, and the rankings of each disease in both search and reported case data were generally stable. Furthermore, the disease rankings in the search data were relatively consistent with the reported case data in each year, with more than 50% of rare diseases having a ranking difference of -20 to 20 between the two systems. In addition, the relationship between the disease rankings in the two systems was generally stable over time. Based on the relationship between the disease rankings in the search and reported case data, rare diseases can be classified into two categories.

**Conclusion:** Online search queries may provide an important new resource for detecting rare diseases. Rare diseases can be classified into two categories to guide different epidemiological research strategies.

**Keywords:** Rare disease; Epidemiological research; Search query; reported case data

**Background**

Rare diseases have become a major public health concern worldwide. In addition to the disease burden, patients with rare diseases often face a lack of treatment options, financial burden, and psychological stress [1]. Currently, there is no consensus on an international definition of rare diseases [2], and the average prevalence threshold for the definition of rare diseases was between 40 and 50 cases/100,000 people based on the analysis of global data [3]. However, despite the low prevalence for each rare disease, the overall population prevalence was estimated to be 3.5–5.9% due to the wide variety of diseases included [4], corresponding to a vast patient population suffering from rare diseases.

Epidemiological research on rare diseases is difficult due to their rarity, scattered distribution, and the influence of the socioeconomic status of different regions on the diagnosis ability of rare diseases [5]. Traditional methods of epidemiological surveys require considerable manpower and time, and are costly and unsuitable for rare diseases. The rare disease direct reporting system is a national database for rare diseases [6], which included anonymized confirmed rare disease cases since 2016 reported by 324 hospitals across China. However, owing to the diagnostic difficulty of rare diseases, which always require complex tests, including metabolite examination, pathological analysis, and genetic tests, the database may contain missing data and delays. In addition, epidemiological studies should be conducted based on the characteristics of different categories of rare diseases. Therefore, it is necessary to classify rare diseases to guide targeted epidemiological research.

With the development of the Internet, online search queries have been used for disease surveillance, which has the advantages of being real-time, having wide coverage, and being low cost. Previous studies have mainly utilized this vast resource to study the epidemiology of communicable diseases, such as influenza [7]; hand, foot and mouth disease [8]; human immunodeficiency virus [9]; measles [10]; conjunctivitis [11]; coronavirus disease (COVID-19) [12]; and some common chronic diseases, such as heart disease [13], gastrointestinal diseases [14], and kidney stones [15]. By analyzing online search behaviors, a high correlation between disease-related queries and officially released data was detected. To date, very few studies have examined the search data in relation to rare diseases.

In this study, we aimed to analyze the relationship between search and reported case data for rare diseases, classify them, and propose an overall epidemiological research strategy for the wide variety of rare diseases.

**Methods**

**Overview of methods**

The methods aimed to compare two sources of rare diseases-related data, online search volume and reported case data, on various rare diseases. In general, we first analyzed the search and reported case data separately to obtain an overview of the disease population and its annual change. Second, we analyzed the relationship between the search data and reported case data, as well as how the relationship changed over time, to help classify rare diseases and propose an overall epidemiological research strategy.

**Range of Rare Diseases**

The National Health Commission and the National Medical Products Administration (NMPA), along with three other authorities, jointly published China’s first “Rare Diseases Catalog” in 2018, which listed 121 rare diseases [6]. Among the 121 diseases, homocysteinemia was not included in the analysis in this study because of the overlap in the definition with another common disease, known as hyperhomocysteinemia.

**Online search data and reported case data**

None of the research procedures involved either individual or private information. Online search data were obtained from the domestic search logs of Sogou, one of the top-3 commercial search engines in China. We generated specific keywords for each rare disease (Table S1), including their disease names, English names, and their specific synonyms and common aliases, based on the guidelines for the diagnosis and treatment of rare diseases (2019) [6]. Then applying full string matching between these keywords and the search queries, we distilled search queries related to rare diseases nationwide from 2016 to 2019, which were called "rare disease related queries." We calculated the ratio of search query numbers for each rare disease to the number of all queries in that year and termed "search popularity." Based on the assumption that search engine users are a random sampling distribution of the population, we multiplied the search popularity by the total population in that year to estimate the number of patients with a certain rare disease, which was termed "search estimated patients." Case data for 120 rare diseases were obtained from China’s national database of rare diseases reported between 2016 and 2019 by 324 hospitals across China.

**Analysis Methods**

We compared the rankings of all rare diseases in each year to explore the relationship between the two kinds of data source. Because rare diseases-related search behaviors are sparse and complex, and the case database still needs improvement, the patient numbers estimated by search data and obtained from case database may contain systemic bias in different years and cannot be compared directly to examine the relationship. Therefore, we calculated annual “disease rankings” in the two systems separately according to the patient numbers for each rare disease. Then, in each year, we grouped every 20 rare diseases according to their search rankings, and calculated their average reported case rankings. We evaluated the consistency between search ranking groups and their average reported case rankings using Pearson’s correlation analysis.

To explore whether the relationship of search and reported case rankings were stable over time, we calculated the annual ranking differences for each disease by subtracting reported case rankings from search rankings, and divided the ranking differences into three groups $\left( -\infty,-20 \right), \left[ -20,20 \right], (20,+\infty)$, which were given IDs 0,1,2, and termed as “ranking difference groups (RDGs)”. We quantitatively investigated the contribution of time to the changes of RDGs by a generalized linear model (GLM). Specifically, years and diseases were used as input categorical variables, and RDG was taken as the dependent variable.

$$RDG\sim C\left( year \right)+C(disease)$$

We fitted the GLM on all data for 120 diseases over four years and reported the contributions of years and diseases to the RDG variable by the coefficients, z values and P values. Statistical analyses and model fitting were conducted using Python3.6 and a Python module statsmodels 0.11.1.

**Results**

**Overview of the comparison between search and reported case data**

In total, 120 rare diseases were included in this study. From 2016 to 2019, the numbers of search estimated patients with rare diseases were 103438, 128521, 117431, and 138002 for each year, and those of the reported patients were 28610, 34360, 41993, and 48264, respectively. Among the 120 rare diseases, 46 (38.3%) had a consistently higher number of search estimated patients during the 4 years, while 37 diseases (30.8%) had consistently higher reported case numbers.

**Ranking of rare diseases in search and reported case data**

In general, the rankings for each disease in the search and reported case data were stable. We took the annual changes of the top 10 high-rates diseases in the search and reported case data as an example to show the relatively stable disease rankings (Fig.1a,b). Seven diseases consistently ranked in the top 10 in the search data and eight diseases consistently ranked in the top 10 in the reported case data.

**Relationship of the disease rankings between search and reported case data**

An overview of the comparisons between rankings in the search and reported case data for 120 rare diseases was presented in Fig.2 and Table 1. In general, the disease rankings in the search data were consistent with the reported case data for each year (Fig.2), with more than 50% of rare diasease had a ranking difference between -20 and 20 (Table 1).

We further analyzed the disease intersections in each two adjacent years for each RDG, and the dominant intersection sizes in Fig.3 suggest that the relationship of disease rankings between the search and reported case data were generally stable over time. We also demonstrated the inter-year stability of the relationship using a GLM, which showed that the time had little effect on the changes of RDGs with small coefficient values (Table 2), compared to the coefficient values of diseases.

Table 1 Ranking differences between the search and reported case data for 120 rare diseases

| Search ranking- reported case ranking | Number of rare diseases | | | |
| --- | --- | --- | --- | --- |
|  | 2016 | 2017 | 2018 | 2019 |
| <-20 | 27 | 29 | 26 | 26 |
| [-20,20] | 68 | 68 | 65 | 68 |
| >20 | 25 | 23 | 29 | 26 |

Table 2 The contribution of time (in terms of years) and diseases to the changes of RDGs by a generalized linear model

| **Variable** | **Coefficient** | **z** | **P>\|z\|** |
| --- | --- | --- | --- |
| C(year)=2016 | -0.0244 | -0.083 | 0.934 |
| C(year)=2017 | -0.0577 | -0.196 | 0.845 |
| C(year)=2018 | 0.0256 | 0.087 | 0.931 |
| C(year)=2019 | -0.0161 | -0.054 | 0.957 |
| C(disease)[group 0]^1^ | -0.821 | -5.427 | 0.014 |
| C(disease)[group 1]^1^ | 0.014 | 0.095 | 0.629 |
| C(disease)[group 2]^1^ | 0.878 | 5.803 | 0.009 |
| Intercept (b) | 1.000 | 3.298 | 0.001 |

^1^ We divided all rare diseases into three groups according to their RDGs in 2016 and reported the average values of each group.

Abbreviations: RDG，ranking difference group.

**Rare disease classification**

According to the generally stable relationship of the disease rankings in the search and reported case data, rare diseases can be classified into two categories. The first category included diseases with a ranking consistency between the search and reported case data, and diseases with high rankings in case data but low rankings in search data. The second category included diseases with high rankings in the search data, but low rankings in the reported case data. It is worth noting that diseases with a ranking difference of -20 to 20 were considered to have consistency between the search and reported case data in this study, the cut-off value of which could be adjusted according to detailed research needs.

**Discussion**

In this study, we analyzed large-scale online search queries for rare diseases and the relationship between search and reported case data to classify rare diseases and explore a new method for epidemiological research on rare diseases. In general, rankings of each rare disease in both the search and reported case data were stable, and rankings in the search data were relatively consistent with the reported case data. In addition, the relationship of disease rankings between the search and reported case data were generally stable over time. According to the relationship between the disease rankings in the two systems, rare diseases can be classified into two categories to guide different epidemiological research strategies.

We found disease ranking to be an appropriate index for studying the relationship between the two systems owing to their stability. After comparing the search data with reported case data, we detected a category of diseases with a ranking consistency between the two systems, as well as diseases with high rankings in the reported case data but low rankings in the search data. For them, we consider that the current national database can provide relatively comprehensive case collection. Particularly, for diseases with a ranking consistency between the search and reported case data, computational models can be constructed to predict the patient numbers of different rare diseases, which would provide a valuable supplement to the national database.

The other category of rare diseases had high rankings in the search data, but low rankings in the reported case data. The first possible reason for these inconsistencies is that disease publicity may influence the patient numbers estimated by search data [16]. Because of the diversity of search populations, some of the search data may come from non-rare disease populations, such as doctors who treat rare diseases or users interested in hot news reports. This leads to an overestimation of disease rates in the search data. The second reason may be that there are potential undiagnosed cases due to the difficulty in diagnosing rare diseases, resulting in under-registration. Considering the diseases shown in Fig.1 as an example, all the diseases ranked consistently high in the search system received wide public attention. Osteogenesis imperfecta patients with atypical symptoms are difficult to diagnose and require genetic testing [17], which may contain missing reported cases. In addition, for diseases with significant changes in the annual rankings in the search data, we need to identify possible contributing factors. For example, Marfan syndrome showed a significant increase in rank to no. 7 in 2019. We consider this to be related to the character in a popular movie ("The Climber") released in 2019, which increased public awareness of the disease. Therefore, for this group of diseases, we need to further analyze the specific reasons for the high search rankings, identify rare disease patients from a variety of search populations through machine learning algorithms, or provide more information for researchers and policymakers to improve the diagnostic ability and provide more medical support for certain rare diseases.

Our study has some limitations. First, this study aimed to classify rare diseases and propose an overall epidemiological research strategy for the wide variety of rare diseases. Thus, we used “disease ranking” which had a good stability and classification performance. However, subsequent epidemiological research on specific rare diseases require more precise statistical indicators, which is our next target. Second, the keywords used for matching rare disease-related queries in this study included disease names, synonyms, and common aliases, which could cover most of the search populations and is suitable for the research needs in this study, but not comprehensive enough for the research on a certain disease. It would be better to add disease-specific keywords, such as gene mutations and medications, in subsequent prediction model construction.

**Conclusions**

Search engine query data are important new resources for epidemiological research on rare diseases. In general, the rankings for each rare disease in both the search and reported case data were stable, and the rankings in the search data were relatively consistent with the reported case data. In addition, the relationship of disease rankings between search and reported case data was found to be generally stable over time. According to the relationship between disease rankings in the two systems, rare diseases can be classified into two categories to guide subsequent epidemiological research strategies. In particular, for diseases with a ranking consistency between the search and reported case data, we consider the current national database to be relatively comprehensive, and we can construct computational models to predict the patient numbers of specified rare diseases. For diseases with high rankings in search data but low rankings in reported case data, we need to further identify real rare disease patients from a variety of search populations through machine learning algorithms, or improve the diagnostic ability and provide more medical support for them.

**List of abbreviations**

NMPA: National Medical Products Administration

RDGs: ranking difference groups

GLM: generalized linear model

**Declarations**

***Ethics approval and consent to participate***

The study was conducted in accordance with the Declaration of Helsinki and approved by the Ethics Committee of Peking Union Medical College Hospital (S-k1790).

***Consent for publication***

Not applicable.

***Availability of data and materials***

The search data that support the findings of this study are available at the following URL: https://github.com/JiayuLi-997/RD_online_search_data. The reported case data used during the current study are available from the administrative group of the national rare disease direct reporting system on reasonable request.

***Competing interests***

The authors declare that they have no competing interests.

***Funding***

This research was funded by Tsinghua University-Peking Union Medical College Hospital Initiative Scientific Research Program (2019ZLH202), the Natural Science Foundation of China (U21B2026), and Tsinghua University Guoqiang Research Institute.

***Authors’ contributions***

L Zhang, Y Jin, and JY Li collected and analyzed the data. ZY He and DD Zhang collected the data. L Zhang wrote the paper. M Zhang, and SY Zhang designed the project. M Zhang revised the manuscript. All authors read and approved the final manuscript.

***Acknowledgements***

Our most sincere appreciation goes to rare disease patients in China. We also appreciate the whole team of China’s national database of rare diseases for their meticulous work.

**References**

1. Li X, Zhang X, Zhang S, Lu Z, Zhang J, Zhou J, et al. Rare disease awareness and perspectives of physicians in China: a questionnaire-based study. Orphanet J Rare Dis. 2021;16:171.

2. Ferreira CR. The burden of rare diseases. Am J Med Genet A. 2019;179:885-92.

3. Richter T, Nestler-Parr S, Babela R, Khan ZM, Tesoro T, Molsen E, et al. International Society for Pharmacoeconomics and Outcomes Research Rare Disease Special Interest Group. Rare disease terminology and definitions-a systematic global review: report of the ISPOR Rare Disease Special Interest Group. Value Health. 2015;18:906-14.

4. Nguengang Wakap S, Lambert DM, Olry A, Rodwell C, Gueydan C, Lanneau V, et al. Estimating cumulative point prevalence of rare diseases: analysis of the Orphanet database. Eur J Hum Genet. 2020;28:165-73.

5. Guo J, Liu P, Chen L, Lv H, Li J, Yu W, et al. National Rare Diseases Registry System (NRDRS): China’s first nation-wide rare diseases demographic analyses. Orphanet J Rare Dis. 2021;16:515.

6. Zhang S, Chen L, Zhang Z, Zhao Y. Orphan drug development in China: progress and challenges. Lancet. 2019;394:1127-8.

7. Ginsberg J, Mohebbi MH, Patel RS, Brammer L, Smolinski MS, Brilliant L. Detecting influenza epidemics using search engine query data. Nature. 2009;457:1012-4.

8. Xu D, Liu Y, Zhang M, Ma S, Cui A, Ru L. Predicting epidemic tendency through search behavior analysis. The twenty-second international joint conference on artificial intelligence, Boston, MA, USA, August 20-23, 2000.

9. Li K, Liu M, Feng Y, Ning C, Ou W, Sun J, et al. Using Baidu search engine to monitor AIDS epidemics inform for targeted intervention of HIV/AIDS in China. Sci Rep. 2019;9:320.

10. Samaras L, Sicilia MA, García-Barriocanal E. Predicting epidemics using search engine data: a comparative study on measles in the largest countries of Europe. BMC Public Health. 2021;21: 100.

11. Deiner MS, McLeod SD, Wong J, Chodosh J, Lietman TM, Porco TC. Google searches and detection of conjunctivitis epidemics worldwide. Ophthalmology. 2019;126:1219-29.

12. Tu B, Wei L, Jia Y, Qian J. Using Baidu search values to monitor and predict the confirmed cases of COVID-19 in China: - evidence from Baidu index. BMC Infect Dis. 2021;21: 98.

13. Senecal C, Widmer RJ, Lerman LO, Lerman A. Association of search engine queries for chest pain with coronary heart disease epidemiology. JAMA Cardiol. 2018;3:1218-21.

14. Hassid BG, Day LW, Awad MA, Sewell JL, Osterberg EC, Breyer BN. Using search engine query data to explore the epidemiology of common gastrointestinal symptoms. Dig Dis Sci. 2017;62:588-92.

15. Breyer BN, Sen S, Aaronson DS, Stoller ML, Erickson BA, Eisenberg ML. Use of Google Insights for Search to track seasonal and geographic kidney stone incidence in the United States. Urology. 2011;78:267-71.

16. Cook AR, Chen MI, Pin Lin RT. Internet search limitations and pandemic influenza, Singapore. Emerg Infect Dis. 2010;16:1647-9.

17. Rauch F, Glorieux FH. Osteogenesis imperfecta. Lancet. 2004;363:1377.

**Figure Legends**

**Fig.1. Annual changes in the top 10 rare diseases in the search and reported case data**

(a) Top 10 high-rates diseases in the search data (b) Top 10 high-rates diseases in the reported case data. Diseases in bold represent those with changes over four years. PAH: pulmonary arterial hypertension.

**Fig.2. Relationship of disease rankings between search and reported case data**

The ordinate values ​​represent the average reported case rankings corresponding to the six groups of diseases ranked 1-20, 21-40, 41-60, 61-80, 81-100, and 101-120 in the search data.

**Fig.3. Disease intersections in two adjacent years**

(a) Diseases with a ranking difference of < -20 between the search and reported case data (b) Diseases with a ranking difference of -20 to 20 between the search and reported case data (c) Diseases with a ranking difference of > 20 between the search and reported case data.
