# Supplementary material for: Epidemiological research on rare diseases using large-scale online search queries and reported case data
Source: Orphanet J Rare Dis. 2023 Aug 9;18:236. doi: 10.1186/s13023-023-02839-7 (PMC10411025; doi:10.1186/s13023-023-02839-7)
Supplement: Supplementary file 2 — Supplementary Material 2 [file 13023_2023_2839_MOESM2_ESM.doc]

**Responses for each of the reviewer’s valued questions and comments**

**Reviewer 1:**

**1) Background**

**- Lines 60-65: This section and the article's results section [3.1 Definition, number, and cumulative prevalence of rare diseases: "Certain diseases are more prevalent in specific regions due to founder mutations and might therefore be formally rare in the majority of countries, but not in others. There is no consensus about an international definition of rare disease; in fact, a systematic review identified 296 definitions from 1,109 organizations across 32 international jurisdictions (Richter et al., 2015). This review found a global average of 40 cases/100,000 people …"] by Ferreira [Ferreira CR. The burden of rare diseases. Am J Med Genet Part A. 2019;179A:885-892. https://doi.org/10.1002/ajmg.a.61124] are too similar. Please rewrite it.**

**R:** Thanks for your kind suggestion. We modified related content in the Background section on pages 5, as below.

*Currently, there is no consensus on an international definition of rare diseases [2], and the average prevalence threshold for the definition of rare diseases was between 40 and 50 cases/100,000 people based on the analysis of global data [3]. However, despite the low prevalence for each rare disease, the overall population prevalence was estimated to be 3.5–5.9% due to the wide variety of diseases included [4], corresponding to a vast patient population suffering from rare diseases.*

**2) Background**

**- Lines 62-63: The paper you cited [Guo J, Liu P, Chen L, et al. National Rare Diseases Registry System (NRDRS): China's first nation-wide rare diseases demographic analyses. Orphanet J Rare Dis. 2021;16(1):515. https://doi.org/10.1186/s13023-021-02130-7] to does not claim that the rare disease definition is influenced by the country's socioeconomic status, but rather that their results are influenced by the fact that in China the two best mapped areas are the richest with the most plentiful healthcare resources healthcare resources ["…These two regions have the most abundant healthcare resources in China. People in these coastal provinces tend to be more informed, wealthier, and more likely to seek diagnosis and care. The high concentration of PIs and researchers in these two regions could also be a contributing factor"]. Please reconsider this sentence.**

**R:** Thanks for your kind suggestion. We modified the content in the second paragraph of the Background section based on the work of Guo J et al. on pages 5, as below.

*Epidemiological research on rare diseases is difficult due to their rarity, scattered distribution, and the influence of the socioeconomic status of different regions on the diagnosis ability of rare diseases [5].*

**3) Background**

**- Line 68: "…The rare disease direct reporting system…". Which one? NRDRS? Please specify which system.**

**R:** Thank you for your question. The rare disease direct reporting system and NRDRS are the two national rare disease databases with different building methods and focuses. The rare disease direct reporting system included data of confirmed cases of rare diseases from more than 300 hospitals across the country since 2016, and NRDRS is an investigator-initiated online rare diseases registry.

We modified the Background section on pages 5, as below. We also replaced the term “registry data” with “reported case data” throughout the manuscript.

*The rare disease direct reporting system is a national database for rare diseases [6], which included anonymized confirmed rare disease cases since 2016 reported by 324 hospitals across China.*

**4) Methods**

**I strongly encourage you to put the methods section after the background. Understanding the paper becomes harder when reading the results without knowing how they were obtained.**

**R**: Thank you for your kind suggestion. We have moved the Methods section after the Background section.

**5) Methods**

**- Could you please elaborate on your methods? Did you use any algorithms?**

**R**: Thank you for your kind suggestion. We have elaborated on the methods on pages 7-10, as below.

***Methods***

***Overview of methods***

*The methods aimed to compare two sources of rare diseases-related data, online search volume and reported case data, on various rare diseases. In general, we first analyzed the search and reported case data separately to obtain an overview of the disease population and its annual change. Second, we analyzed the relationship between the search data and reported case data, as well as how the relationship changed over time, to help classify rare diseases and propose an overall epidemiological research strategy.*

***Range of Rare Diseases***

*The National Health Commission and the National Medical Products Administration (NMPA), along with three other authorities, jointly published China’s first “Rare Diseases Catalog” in 2018, which listed 121 rare diseases [6]. Among the 121 diseases, homocysteinemia was not included in the analysis in this study because of the overlap in the definition with another common disease, known as hyperhomocysteinemia.*

***Online search data and reported case data***

*None of the research procedures involved either individual or private information. Online search data were obtained from the domestic search logs of Sogou, one of the top-3 commercial search engines in China. We generated specific keywords for each rare disease (Table S1), including their disease names, English names, and their specific synonyms and common aliases, based on the guidelines for the diagnosis and treatment of rare diseases (2019) [6]. Then applying full string matching between these keywords and the search queries, we distilled search queries related to rare diseases nationwide from 2016 to 2019, which were called "rare disease related queries."* *We calculated the ratio of search query numbers for each rare disease to the number of all queries in that year and termed "search popularity." Based on the assumption that search engine users are a random sampling distribution of the population, we multiplied the search popularity by the total population in that year to estimate the number of patients with a certain rare disease, which was termed "search estimated patients." Case data for 120 rare diseases were obtained from China’s national database of rare diseases reported between 2016 and 2019 by 324 hospitals across China.*

***Analysis Methods***

*We compared the rankings of all rare diseases in each year to explore the relationship between the two kinds of data source. Because rare diseases-related search behaviors are sparse and complex, and the case database still needs improvement, the patient numbers estimated by search data and obtained from case database may contain systemic bias in different years and cannot be compared directly to examine the relationship. Therefore, we calculated annual “disease rankings” in the two systems separately according to the patient numbers for each rare disease. Then, in each year, we grouped every 20 rare diseases according to their search rankings, and calculated their average reported case rankings. We evaluated the consistency between search ranking groups and their average reported case rankings using Pearson’s correlation analysis.*

*To explore whether the relationship of search and reported case rankings were stable over time, we calculated the annual ranking differences for each disease by subtracting reported case rankings from search rankings, and divided the ranking differences into three groups**, which were given IDs 0,1,2, and termed as “ranking difference groups (RDGs)”. We quantitatively investigated the contribution of time to the changes of RDGs by a generalized linear model (GLM). Specifically, years and diseases were used as input categorical variables, and RDG was taken as the dependent variable.*

*We fitted the GLM on all data for 120 diseases over four years and reported the contributions of years and diseases to the RDG variable by the coefficients, z values and P values. Statistical analyses and model fitting were conducted using Python3.6 and a Python module statsmodels 0.11.1.*

**6)** **Methods**

**- What keywords did you choose? Could you include it as supplementary material?**

**R**: Thank you for your question. We generated keywords for each rare disease, including their disease names, English names, and their specific synonyms and common aliases, based on the guidelines for the diagnosis and treatment of rare diseases (2019) (Methods, page 8). The keywords for each rare disease were listed in Supplementary table 1.

**7) Methods**

**- What are the 2 commercial search engines? Google? Local? Are they international or domestic in environment?**

**R:** Sorry for the unclear introduction of the search engine. Since we focused on the rare disease epidemiological research in China, we used the domestic search logs of Sogou, one of the top-3 commercial search engines in China. We modified related content in the Methods section on page 8, as below.

*Online search data were obtained from the domestic search logs of Sogou, one of the top-3 commercial search engines in China.*

**8) Results**

**- In the results section, overall, you must include what was found. In your paper, there is some overlap among methods, results and discussion [i.e., lines 94-101 describe a method and a study limitation].**

**R**: Thank you for your kind suggestion. We integrated the methodological content in the Results section into the Methods section, and modified related content in the Methods on pages 8-9, and Results on page 10, as below.

***Methods：***

***Analysis Methods***

*We compared the rankings of all rare diseases in each year to explore the relationship between the two kinds of data source. Because rare diseases-related search behaviors are sparse and complex, and the case database still needs improvement, the patient numbers estimated by search data and obtained from case database may contain systemic bias in different years and cannot be compared directly to examine the relationship. Therefore, we calculated annual “disease rankings” in the two systems separately according to the patient numbers for each rare disease.*

***Results：***

***Ranking of rare diseases in search and reported case data***

*In general, the rankings for each disease in the search and reported case data were stable. We took the annual changes of the top 10 high-rates diseases in the search and reported case data as an example to show the relatively stable disease rankings (Fig.1a,b). Seven diseases consistently ranked in the top 10 in the search data and eight diseases consistently ranked in the top 10 in the reported case data.*

**9) Results**

**- Furthermore, you claim that the "disease ranking in the search data was relatively consistent with the registry data for each year"; how do you support this finding? Is there a statistically significant difference? If so, please report it.**

**R:** Thank you for your kind suggestion. We added the statistical methods in the Methods section on page 9, and reported the analysis results (including r and *P* values) in Fig 2, as below.

***Methods：***

*Then, in each year, we grouped every 20 rare diseases according to their search rankings, and calculated their average reported case rankings. We evaluated the consistency between search ranking groups and their average reported case rankings using Pearson’s correlation analysis.*

***Fig.2. Relationship of disease rankings between search and reported case data***

*The ordinate values represent the average reported case rankings corresponding to the six groups of diseases ranked 1-20, 21-40, 41-60, 61-80, 81-100, and 101-120 in the search data.*

**10) Results**

**- As well as, if you affirm that "the relations of disease ranking between the search and registry data is stable overtime", how do you demonstrate this? Have you conducted a group comparison? regression? other?**

**These findings are currently lacking; please review the results and attempt to justify them using the appropriate statistical analysis.**

**R**: Thank you for your kind suggestion. We added the statistical methods in the Methods section on page 9, and reported the analysis results to demonstrated the inter-year stability of the relationship in the Results section on page 11 and Table 2, as below.

***Methods:*** *To explore whether the relationship of search and reported case rankings were stable over time, we calculated the annual ranking differences for each disease by subtracting reported case rankings from search rankings, and divided the ranking differences into three groups**, which were given IDs 0,1,2, and termed as “ranking difference groups (RDGs)”. We quantitatively investigated the contribution of time to the changes of RDGs by a generalized linear model (GLM). Specifically, years and diseases were used as input categorical variables, and RDG was taken as the dependent variable.*

*We fitted the GLM on all data for 120 diseases over four years and reported the contributions of years and diseases to the RDG variable by the coefficients, z values and P values. Statistical analyses and model fitting were conducted using Python3.6 and a Python module statsmodels 0.11.1.*

***Results:*** *We also demonstrated the inter-year stability of the relationship using a GLM, which showed that the time had little effect on the changes of RDGs with small coefficient values (Table 2), compared to the coefficient values of diseases.*

***Table 2*** The contribution of time (in terms of years) and diseases to the changes of RDGs by a generalized linear model

| ***Variable*** | ***Coefficient*** | ***z*** | ***P>\|z\|*** |
| --- | --- | --- | --- |
| *C(year)=2016* | *-0.0244* | *-0.083* | *0.934* |
| *C(year)=2017* | *-0.0577* | *-0.196* | *0.845* |
| *C(year)=2018* | *0.0256* | *0.087* | *0.931* |
| *C(year)=2019* | *-0.0161* | *-0.054* | *0.957* |
| *C(disease)[group 0]1* | *-0.821* | *-5.427* | *0.014* |
| *C(disease)[group 1]1* | *0.014* | *0.095* | *0.629* |
| *C(disease)[group 2]1* | *0.878* | *5.803* | *0.009* |
| *Intercept (b)* | *1.000* | *3.298* | *0.001* |

*^1^ We divided all rare diseases into three groups according to their RDGs in 2016 and reported the average values of each group.*

*Abbreviations: RDG，ranking difference group.*
